# Composition and the predicted functions of fungal communities and the key drivers in acidic soils of Jiaodong Peninsula, China

**DOI:** 10.3389/fmicb.2024.1496268

**Published:** 2025-01-06

**Authors:** Jing Liu, Zafran Gul Wazir, Guoqin Hou, Guizhen Wang, Fangxu Rong, Yuzhi Xu, Kai Liu, Mingyue Li, Aiju Liu, Hongliang Liu, Hongwen Sun

**Affiliations:** ^1^School of Agricultural Engineering and Food Science, Shandong University of Technology, Zibo, China; ^2^School of Resources and Environmental Engineering, Shandong University of Technology, Zibo, China; ^3^School of Life Sciences and Medicine, Shandong University of Technology, Zibo, China; ^4^Ministry of Education Key Laboratory of Pollution Processes and Environmental Criteria, College of Environmental Science and Engineering, Nankai University, Tianjin, China

**Keywords:** acidic soils, fungal community composition, fungal functional guilds, soil pH, plant pathogens

## Abstract

**Introduction:**

Soil acidification imperils soil health and hinders the agricultural sustainability. As being more tolerant than bacteria to soil acidification, so it would be more meaningful for agricultural management and crop yield to characterize fungal community in acidic soils and manifest its key drivers.

**Method:**

This study investigated the composition and diversity of fungal communities and its key driving factors by collecting 90 soil samples from the acidic region of Jiaodong Peninsula China, spanning 3 × 10^4^ km^2^.

**Results:**

The results indicated that most soil pH values ranged from 5.01 to 6.42, and the exchangeable acidity (E_A_) content raised significantly (*p* < 0.01) along with soil acidic degree increasing. However, no significant differences were found in fungal community diversity and composition among various soil samples, which were all predominantly habited with the phyla of Ascomycota and Basidiomycota. Results of the linear discriminant analysis effect size (LEfSe) analysis revealed that saprophytic fungi were biomarkers of the slightly acidic soil (pH 6.0–6.5), including *Nectriaceae, Thielavia, Nectria, Haematonectria*, and unclassified *Microascaceae*, while plant pathogenic fungi, such as Didymellaceae, were biomarkers of the soils pH < 5.5. Similarly, the FUNGuild results also indicated that saprophytic fungi and pathogenic fungi were the dominant functional guilds in the investigated acidic soils, accounting for 66% of the total fungal communities. Redundancy analysis (RDA) revealed that soil pH as well as nitrate nitrogen (${\text{NO}}_{3}^{-}$-N) and total nitrogen (TN) significantly associated with fungal community at the phylum level, whilst soil pH was the only factor significantly linked to individual fungal classes (*p* < 0.01 or 0.05). The further Mantel test analysis and structural equation modeling (SEM) suggested that, in contrast to the negative and directive driving of soil pH on fungal communities' variation, the specific plant pathogenic fungi, Gibberella and Didymellaceae, were significantly and positively associated with soil acidic characteristics (*p* < 0.05).

**Discussion:**

These findings highlighted that, in addition to modulating the variation of soil fungal community, soil acidification might prime some plant pathogens development. So that, more attentions should be focused on impact of soil acidification on fungal ecology, as well as plant pathogenic fungi.

## 1 Introduction

Acidic soils widely distribute and occupy more than 50% of the world's potentially arable land (Kochian et al., 2005; Ma and Ryan, 2010). In China, the pH of farmland surface soil declined by 0.5 units on average due to excessive application of nitrogen (N) fertilizer during past three decades (Guo et al., 2010). Undoubtedly, soil acidification had become an serious issue in agricultural production in China (Li et al., 2017), as it could result in the significant losses of base cations and nutrients and decrease of cation exchange capacity (CEC), and inhibiting soil microbial activity as well as releasing of aluminum (Al) and manganese (Mn) (Bolan et al., 2003; Kunhikrishnan et al., 2016).

Soil fungi are massively abundantly distributed in soils, and play instrumental roles in the ecosystem, including pedogenesis, nutrient cycling, and disease suppression, etc. (Bardgett and van der Putten, 2014). Moreover, in contrast to soil bacteria, soil fungi exhibit a wider range in C:N and soil pH-growth optimum (Strickland and Rousk, 2010) and are more tolerant to acidic soil environments (Peñalva et al., 2008; Mosier et al., 2016). Recently, several studies investigated the composition and driving factors of soil fungal communities in acidic soils (Pan et al., 2015; Shi et al., 2018; Ye et al., 2020), and widely suggested that soil pH is the key factor modulating the fungal community structures in acidic soils (Muneer et al., 2021; Hu et al., 2024). However, given their prominent roles in key soil processes, more information about the fungal community composition and function in various acidic soils is of primary importance to prioritize ecosystem-level conservation and management efforts.

Previous studies showed that soil fungal communities not only facilitated the decomposition of soil organic matter (SOM) by secreting various degrading enzymes (Chen et al., 2014), but also could establish symbiotic or pathogenic relationships with plants (Pascale et al., 2020; Cao et al., 2024). So that, they were proved to be essential to maintain a variety of functions in above- and belowground ecosystems (Egidi et al., 2019; Yang et al., 2019; Pérez-Izquierdo et al., 2021). Within three trophic modes, FUNGuild classifies fungal community data into more manageable ecological guilds [e.g., arbuscular mycorrhizal (AM) fungi, ectomycorrhizal (ECM) fungi, plant pathogens, wood saprotrophs, etc.], totaling 12 guilds (Nguyen et al., 2016). For example, saprophytic fungi can decompose litter and promote nutrient cycling using their extracellular enzymes for providing essential nutrients for plant growth (Cao et al., 2022). Symbiotic fungi, such as AM fungi and ECM fungi, form associations with plants and help plants absorb nutrients, enhancing their ability to resist abiotic stresses (Cairney, 2012; Wahab et al., 2023). In contrast, plant pathogenic fungi, including *Fusarium, Verticillium*, and *Ilyonectri*, can cause plant diseases by inhibiting root development and causing root rot (De Coninck et al., 2015). Therefore, it is important to understand the structure and function of fungal communities in acidic soils and the potential risk to crop health, beside of their communities' diversity.

Several studies investigated soil microbial communities, including the composition and driving factors of soil fungal communities in acidic soil (Pan et al., 2015; Shi et al., 2018; Ye et al., 2020). Nonetheless, few reported studies have focused on the ecological functions of fungi, especially for those with potential pathogenicity in acidic soils. In this study, the composition and predicting functions of soil fungal communities were investigated in acidic soils of the Jiaodong Peninsula, a typical acidic region of Northern China. We aimed to explore the effect of soil acidification on soil fungal communities' composition and their ecological functions, and decipher their key driving factors, which is significant to assess the ecological risk of soil acidification to agroecosystems. It was hypothesized that: (1) as the similar agricultural practices, variation in the diversity and composition of fungal communities were not significant in various acidic soils of Jiaodong Peninsula, even with distinct acidification degree; (2) soil pH be the key factors driving changes on fungal community composition and ecological functions, as well as the fungal plant pathogens.

## 2 Materials and methods

### 2.1 Site description and soil sampling

**Figure 1:**
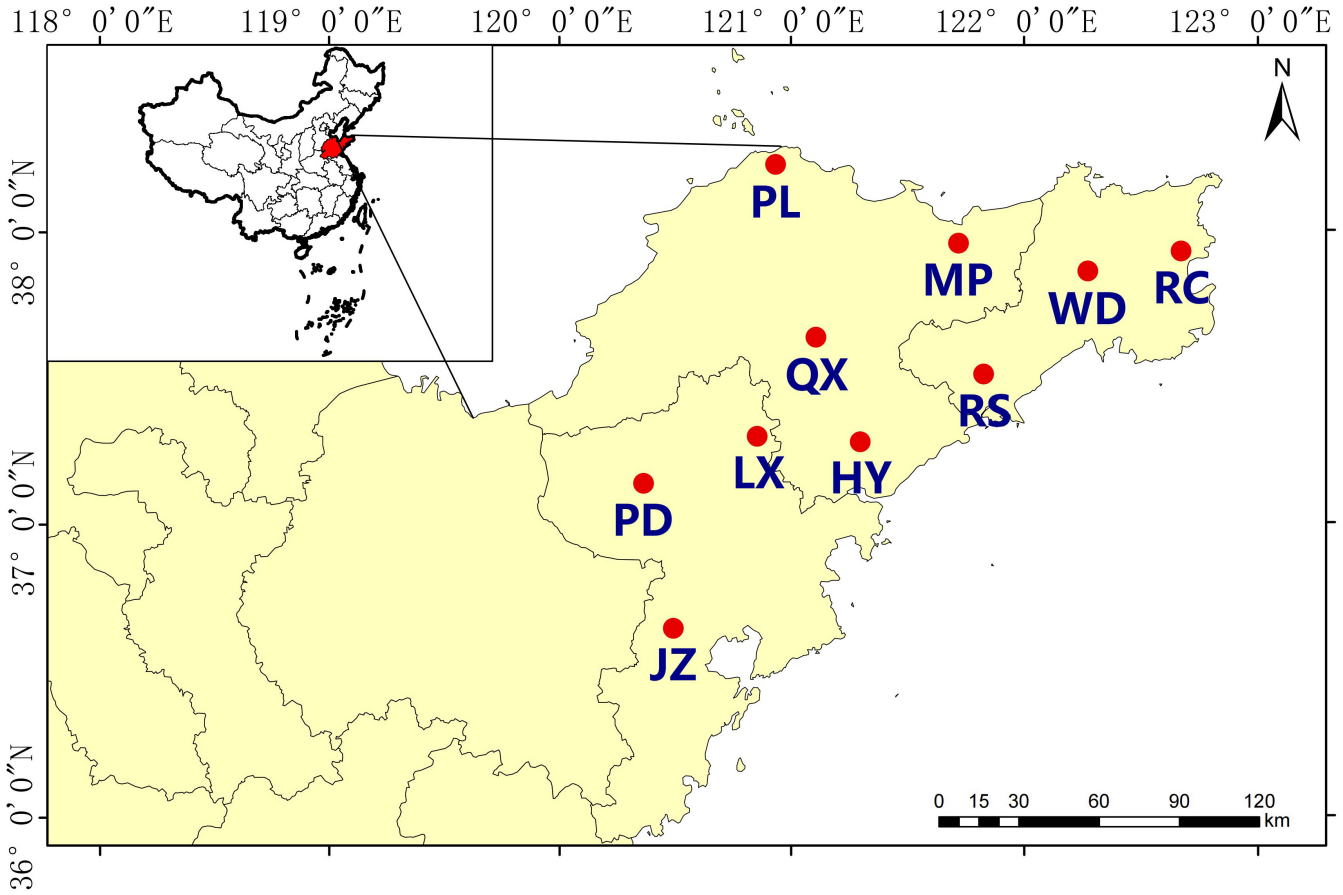
Sampling sites of acidic soils in Jiaodong Peninsula. The abbreviation in the figure refers to the site where samples were collected including Muping (MP), Haiyang (HY), Qixia (QX), Penglai (PL), Jiaozhou (JZ), Pingdu (PD), Laixi (LX), Rongcheng (RC), Rushan (RS), and Wendeng (WD).

Soil samples were collected from10 districts in Qingdao (QD), Yantai(YT), and Weihai (WH) of Jiaodong Peninsula, Shandong Province, China (35°35′-38°23′N, 119°59′-122°71′E, Figure 1). This region features a warm, temperate monsoon continental climate, with mean annual temperatures averaging 12.0–12.6°C. The annual mean rainfall ranges from 650 to 850 mm. The soil type was mainly classified as Alfisols, comprising over 50% (Soil Survey Staff, 2014; Zhao et al., 2023). A sampling point was set around per 7,000 hm^2^ according the area of the selected districts, and a total of 30 sampling points were set up spanning 3 × 10^4^ km^2^ (Figure 1). Three sampling sites were chosen in each district, and at each sampling site, three 200 × 200 m quadrats with relatively similar conditions were selected. The distance between each quadrat was >25 km, thus the quadrats were considered independent from each other. A total of 90 topsoil (0–20 cm) samples were taken from fields in March to May, 2021. All of the soil samples were sieved (2 mm), after removing weeds, fine straw and other debris. A portion of each sample was taken for characterization of physical and chemical properties, while the other was frozen at −80°C for DNA extraction and PCR amplification analysis.

### 2.2 Measurement of soil properties

Soil pH was measured in a soil:deionized water mixture (1:5) by a pH meter. Soil CEC was determined based on the hexaamminecobalt trichloride method (Ciesielski and Sterckeman, 1997). SOM was analyzed by the potassium dichromate colorimetric method (Yang F. et al., 2017). Exchangeable acidity (E_A_), exchangeable hydrogen (E_H+_) and exchangeable aluminum (E_Al_) of the soil samples were performed using a titrator to adjust pH to the objective value in 1.0 M KCl extractions (Chen et al., 2011). Soil leachable ammonium nitrogen (${\text{NH}}_{4}^{+}$-N) and nitrate nitrogen (${\text{NO}}_{3}^{-}$-N) were measured by a UV-visible spectrophotometer in the same extracts (Li C. Y. et al., 2020). Total nitrogen (TN) was digested with the potassium persulfate catalyst and determined in the same analyzer (Cheng et al., 2021). Total phosphorus (TP) was measured by molybdenum-blue colorimetry (Wang et al., 2009). Available phosphorus (AP) content was determined based on the NaHCO_3_ extraction-molybdenum antimony anti-colorimetric method (Shen et al., 2018).

### 2.3 DNA extraction

Soil DNA from different samples were extracted with a FastDNA Spin Kit (MP Biomedicals, Santa Ana, CA) according to the manufacturer's instructions. Extractions were performed in triplicates for each of the selected 26 samples, and the extracts were then pooled for further analysis. The extracted DNA was eluted in 50 μL of Elution buffer and stored at −80°C until measurement in the PCR.

### 2.4 Sequencing and data processing

The fungal ITS2 region was amplified with the primes ITS1FI2 (5′-GTGARTCATCGAATCTTTG-3′) and ITS2 (5′-TCCTCCGCTTATTGATATGC-3′) (Karlsson et al., 2014). PCR amplification was performed in a total volume of 25 μL reaction mixture containing 25 ng of template DNA, 12.5 μL PCR Premix, 2.5 μL of each primer, and PCR-grade water to adjust the volume. The conditions for amplification were 98°C for 30 s; 32 cycles of denaturation at 98°C for 10 s, annealing at 54°C for 30 s, and extension at 72°C for 45 s; and then final extension at 72°C for 10 min. The PCR products were confirmed with 2% agarose gel electrophoresis. Then the PCR products were purified by AMPure XT beads (Beckman Coulter Genomics, Danvers, MA, USA) and quantified by Qubit (Invitrogen, USA). The amplicon pools were prepared for sequencing and the size and quantity of the amplicon library were assessed on Agilent 2100 Bioanalyzer (Agilent, USA) and with the Library Quantification Kit for Illumina (Kapa Biosciences, Woburn, MA, USA), respectively. The libraries were sequenced on NovaSeq PE250 platform at LC-Bio Technology (Hang Zhou, China). The sequencing data have been archived in the NCBI Sequence Read Archive under accession number PRJNA1045351.

Quality filtering on the raw reads were performed under specific filtering conditions to obtain the high-quality clean tags according to the fqtrim (v0.94). Chimeric sequences were filtered using Vsearch software (v2.3.4). After de-replication using DADA2, we obtained amplicon sequence variants (ASVs). The α-diversity and β-diversity were calculated by QIIME2, and the relative abundance (X fungi count/total count) is used in fungi taxonomy. The rarefaction curves of α-diversity indices, including Chao richness, Shannon index and Good's coverage, were saturated based on the ASV information (Supplementary Figure S1). According to the coverage values were 1.00, the majority of soil microbial associations in soil samples were covered. ASVs functional groups were predicted using FUNGuild (https://github.com/UMNFuN/FUNGuild) (Nguyen et al., 2016).

### 2.5 Statistical analysis

Mean and standard deviation of soil properties were calculated by Excel. Soil properties and the α-diversity indices were tested by one-way analysis of variance (ANOVA). Their differences among the soil samples were examined by the least significant difference (LSD) test (*p* < 0.05). These analyses were performed in SPSS 22.0. Liner regression model, columns, and box-plots were created by Origin 2022. Heatmap and correlation network, circos and sankey diagram were performed at the OmicStudio platform (https://www.omicstudio.cn/tool). The β-diversity was assessed through the non-metric multidimensional scaling (NMDS) analysis and the analysis of similarities (ANOSIM) based on Bray-Curtis distances, conducted with the R (version 4.3.2) package “vegan” and visualized using “ggplot2”. To conduct an analysis of differences in microbial communities, we used ALDEx2 analysis and LEfSe analysis for comparison. The ALDEx2 tool was used to determine significant differences at ASV level in terms of relative abundance between pairwise comparisons of fungal communities in soils with different soil pH gradients. Which tool performed the Wilcoxon's rank-sum tests, followed by the Benjamini-Hochberg FDR correction on *p*-value for each feature (Nearing et al., 2022). The linear discriminant analysis (LDA) together with effect size analysis (LEfSe) to identify fungal biomarkers characterizing the different pH range soil sample groups. The redundancy analysis (RDA) was performed using the same package in R, to identify the relationship between fungal community structure and physicochemical properties of the soils.

Based on maximum likelihood estimation, structural equation model (SEM) used IBM SPSS AMOS 24.0 to analyze the direct or indirect effects of soil properties on fungal communities. Several model fit summaries were used to evaluate our model: non-significant chi-square test (*P* > 0.05), low χ^2^/df (< 2), high goodness-of-fit index GFI > 0.9, and low root mean square errors of approximation RMSEA < 0.05.

## 3 Results

### 3.1 Soil physicochemical properties

**Figure 2:**
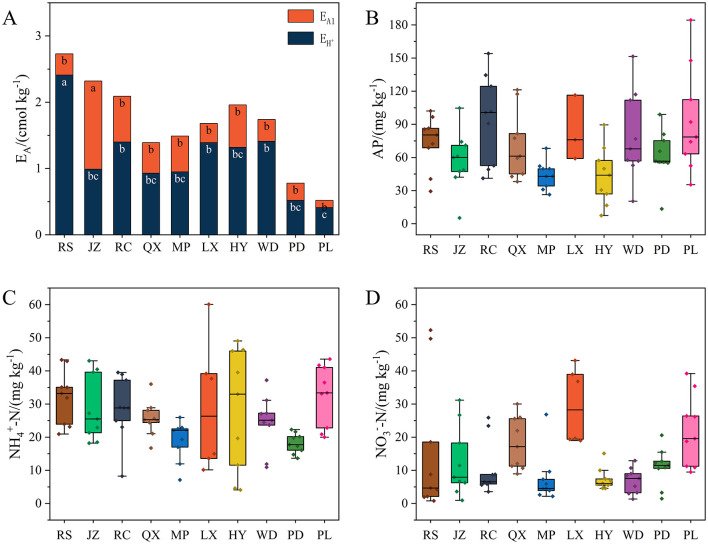
Contents of E_A_ **(A)**, AP **(B)**, ${\text{NO}}_{3}^{-}$-N **(C)**, and ${\text{NH}}_{4}^{+}$-N **(D)** in soil samples. Different lowercase letters denote significant difference among soil sampling sites (*p* < 0.05).

The characteristics of soil acidification in Jiaodong Peninsula were clearly exhilarated in Table 1 and Figure 2A. RS soils had the lowest soil pH value of 5.01, while PL had the highest value (6.42), with the greatest difference of 1.41 units. Accordingly, the contents of E_A_ and ${\text{E}}_{{\text{H}}^{+}}$ in soil were significantly higher at RS than the other sites (*p* < 0.05), whilst the E_Al_ contents in JZ soils were the highest among all the soil samples (*p* < 0.05). Whatever, the significantly negative correlation (*p* < 0.01) between the content of E_A_ and soil pH was also corroborated in the present study (Supplementary Figure S2).

**Table 1:** Basic physicochemical properties of soil samples.

**Site**	**pH (H_2_O)**	**CEC (cmol kg^−1^)**	**SOM (g kg^−1^)**	**TN (g kg^−1^)**	**TP (g kg^−1^)**	**Soil texture (%)**		
						**Sand**	**Silt**	**Clay**
RS	5.01 ± 0.35c	2.32 ± 1.37de	12.37 ± 1.10a	20.14 ± 13.05a	0.43 ± 0.12bc	72.06 ± 7.22ab	22.36 ± 5.78ef	5.59 ± 1.44ef
JZ	5.24 ± 0.49c	5.33 ± 1.80cd	5.19 ± 0.23c	2.00 ± 0.22c	0.32 ± 0.07c	40.89 ± 5.74f	47.29 ± 4.59a	11.82 ± 1.15a
RC	5.39 ± 0.55c	3.18 ± 0.66de	13.09 ± 2.72a	15.17 ± 3.72b	0.78 ± 0.37a	76.44 ± 8.17a	18.84 ± 6.53f	4.71 ± 1.63f
QX	5.58 ± 0.31c	4.42 ± 1.96cd	6.52 ± 0.39bc	2.62 ± 0.56c	0.47 ± 0.11bc	62.89 ± 12.25bcd	29.69 ± 9.80cde	7.42 ± 2.45cde
MP	5.70 ± 0.62abc	0.95 ± 1.03e	7.33 ± 1.10b	11.18 ± 6.49b	0.46 ± 0.28bc	67.67 ± 5.58abc	25.87 ± 4.47def	6.47 ± 1.12def
LX	5.76 ± 1.22abc	8.92 ± 6.25ab	5.12 ± 0.51c	2.47 ± 0.80c	0.51 ± 0.13bc	68.72 ± 3.38abc	25.02 ± 2.71def	6.26 ± 0.68def
HY	5.88 ± 1.08ab	3.29 ± 1.66de	6.59 ± 0.38bc	3.20 ± 0.97c	0.41 ± 0.19bc	53.78 ± 14.58de	36.98 ± 11.67bc	9.24 ± 2.92bc
WD	6.01 ± 0.63ab	4.21 ± 0.89cde	12.86 ± 3.29a	11.21 ± 3.00b	0.55 ± 0.30b	62.50 ± 10.66bcd	30.00 ± 8.53cde	7.50 ± 2.13cde
PD	6.01 ± 0.68ab	11.53 ± 4.55a	5.56 ± 0.44bc	2.08 ± 0.39c	0.32 ± 0.11bc	61.11 ± 17.20cd	31.11 ± 13.76cd	7.78 ± 3.44cd
PL	6.42 ± 0.71a	7.17 ± 4.85bc	6.44 ± 0.50bc	3.80 ± 1.83c	0.45 ± 0.16bc	46.94 ± 5.70ef	42.44 ± 4.56ab	10.61 ± 1.14ab

CEC, cation exchange capacity; SOM, soil organic matter; TN, total nitrogen; TP, total phosphorus. All values are reported as mean ± SDs, *n* = 3. Different lowercase letters indicate significant difference at *p* < 0.05 among the samples.

In most of soil samples, the nitrogen availability was usually lower with the increase of soil acidity (Figures 2C, D), while the contents of TN followed the opposite trend. The AP and TP contents were the highest in soils sampled from RC, which was consistent to the serious acidic conditions of this site. However, no significant associations were found between soil acidification gradient and the other soil properties, such as the SOM contents were significantly higher in soils from RC, RS and WD than the other sites (*p* < 0.05) (Figure 2B), whilst the contents of CEC in MP soils were the lowest among all the soil samples (*p* < 0.05), indicating the soils in MP had lower supply capacity of nutrients than the other sampling sites. There was no obvious fluctuation in the soil texture for most soil samples which predominant component was all of sand.

### 3.2 Characteristics of fungal community structure

**Figure 3:**
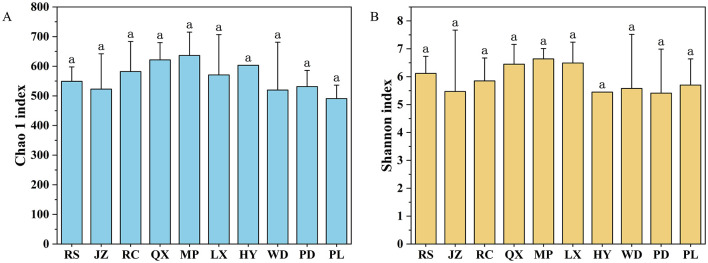
α-diversity indices of fungal communities in farmland: **(A)** Chao richness, and **(B)** Shannon index.

**Figure 4:**
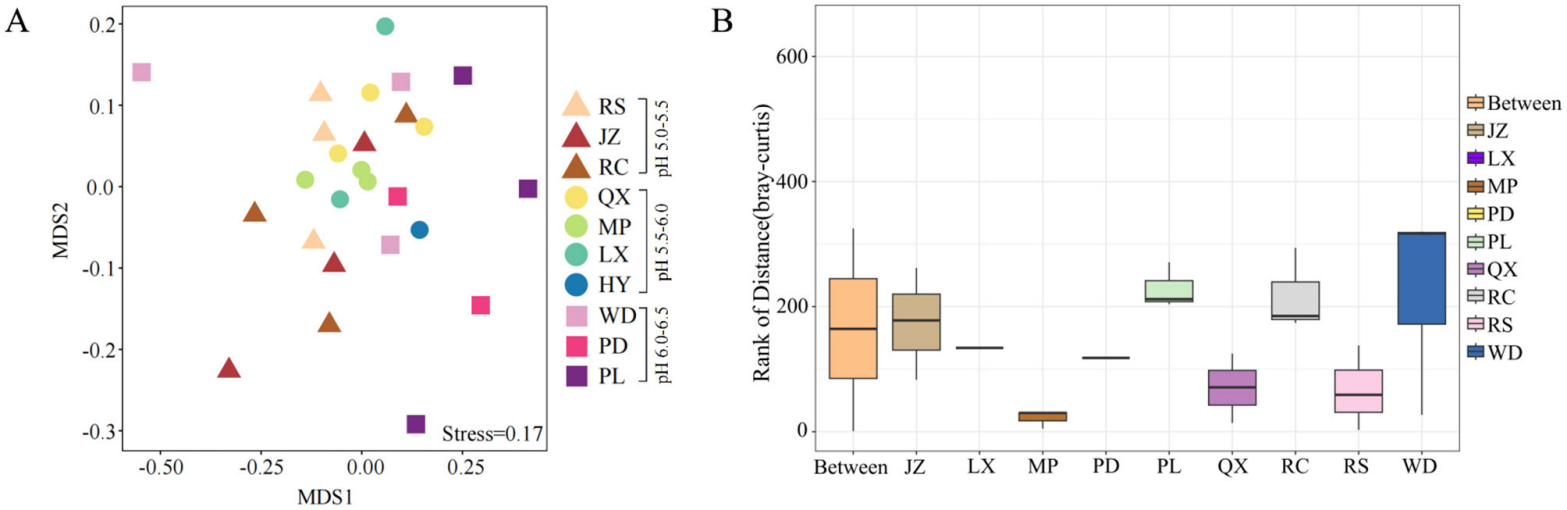
β-diversity of fungal communities in farmland: **(A)** non-metric multidimensional scaling (NMDS) plot, and **(B)** the analysis of similarities (ANOSIM) at the ASV level. The box corresponding to the “between” represents the distance value of the difference among groups, while the other boxes represent the distance value of the difference within each group.

Among all the investigated soils, no significant difference was found in fungal diversity, including Chao richness and Shannon index, even they were under various soil acidification degree (Figures 3A, B). Moreover, the NMDS analysis revealed that fungal communities did not exhibit a distinct separation along the soil pH gradient on the NMDS axis 1 (Figure 4A, Stress = 0.17). The NMDS results based on Bray-Curtis distance showed that the β-diversity of soil fungal communities was similar in Jiaodong Peninsula (Figure 4A), which were also substantiated by the results of ANOSIM (*p* > 0.05) (Figure 4B). The ALDEx2 analysis revealed that no significant difference was found in fungal community composition under various soil pH gradients (Supplementary Figure S6). This result was consistent with the fungal community diversity with increased soil acidity. All of these results suggested that fungal communities were every tolerant to acidic soils.

**Figure 5:**
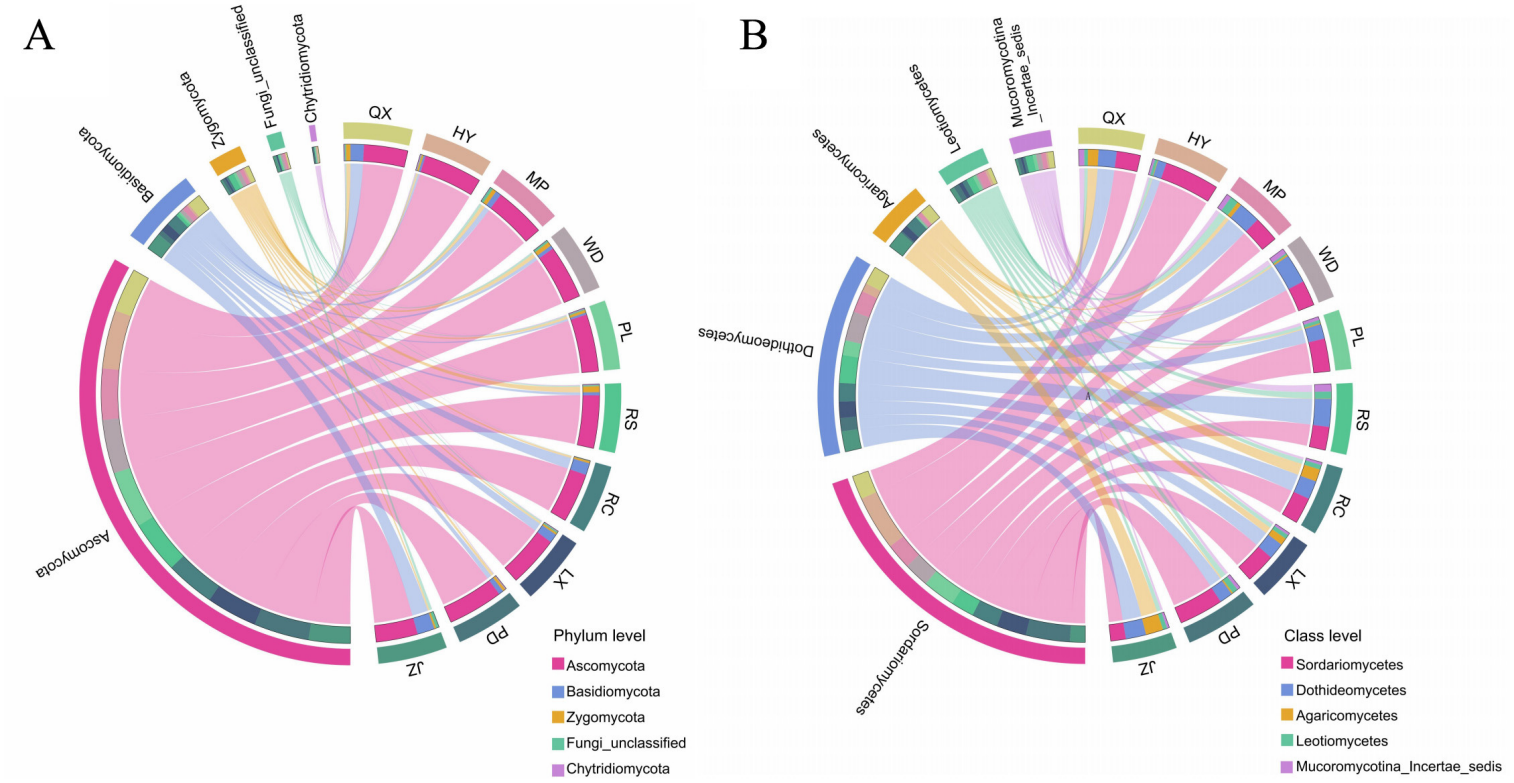
Composition of the top 5 soil fungi at phylum level **(A)** and class level **(B)**.

The top five fungi with the largest relative abundance at the phylum level and class level were selected for the comparative analysis of fungal community composition (Figures 5A, B). Soil fungal communities in Jiaodong Peninsula primarily included members of phyla *Ascomycota* (65.24–91.60%), *Basidiomycota* (3.24–27.17%), *Zygomycota* (2.60–9.52%), unclassified Fungi (0.44–4.56%) and *Chytridiomycota* (0.16–1.55%), accounting for 99.88–100.00% of all the identified phyla. The first dominant phylum was *Ascomycota*, which presented a higher relative abundance in slightly acidic soils compared to strongly acidic soils, however *Basidiomycota* exhibited an inverse trend. The predominant classes in all soil samples were *Dothideomycetes* and *Sordariomycetes*, and in HY and PD soils, *Sordariomycetes'* relative abundance even exceeded 50% of all the designed classes. However, *Dothideomycetes'* relative abundance in WD and RS soils was 3.4–and 3.2 - fold higher than that in HY soil, respectively. As shown on Figures 5A, B, the relative abundance of the dominant phyla and classes in acidic soils exhibited spatial variations with increased soil acidity. Further analysis of the top 10 genera (relative abundance > 0.01%) revealed that members of *Ascomycota, Basidiomycota*, and *Zygomycota* were shared by all the soil samples (Supplementary Figure S3). The most abundant genera (relative abundance > 1%) were *Pseudogymnoascus* and *Mortierella*, followed by *Gibberella, Humicola*, and *Podospora*. The relative abundance of *Humicola* in HY soils had the highest value (43.94), significantly surpassing that of other soils, and this predominance may have been unrelated to soil acidity. In addition, the most representative pathotroph genera, including *Gibberella*, unclassified *Didymellaceae, Oidiodendron* and *Pseudogymnoascus*, were also determined among all soil samples. The RC soil exhibited the highest relative abundance of *Pseudogymnoascus* at 12.48%, which was classified as strongly acidic soil, whereas the PL soil, characterized as slightly acidic, showed the lowest relative abundance at 2.63%. Within the RS soil, the relative abundance of the unclassified *Didymellaceae* was the highest, reaching 15.72%, while the relative abundance of PL was the lowest, recorded at 0.21%. Therefore, the pathogenic fungi exhibited significant spatial distribution differences across soils with varying degrees of soil acidification, suggesting that soil pH likely played a pivotal role in shaping the composition and dominance of fungal communities within these ecosystems.

The LEfSe algorithm with an LDA score of 3.5 was used to characterize the differences in soil samples of different pH ranges. The results showed that 30 fungal clades exhibited significant differences in all soil samples (Supplementary Figure S4). Specifically, the less acidic soils (pH 6.0–6.5) were rich in *Nectriaceae* (family), *Thielavia* (genus), *Nectria* (genus), *Haematonectria* (genus) and unclassified *Microascaceae* (genus), which were belonged to the phylum *Ascomycota*. While in the higher acidic soil pH (pH 5.0–5.5), *Didymellaceae* with the highest abundance was only the biomarker taxa (family), yet belonging to *Ascomycota*. Besides, the enriched fungal taxa in pH 5.5–6.0 soils were all affiliated with the phyla *Ascomycota* (from class to genus) and *Basidiomycota* (from family to genus), but there were exhibited spatial variations in taxon abundance along soil pH gradients.

### 3.3 Relationship between environmental characteristics and fungal communities

**Figure 6:**
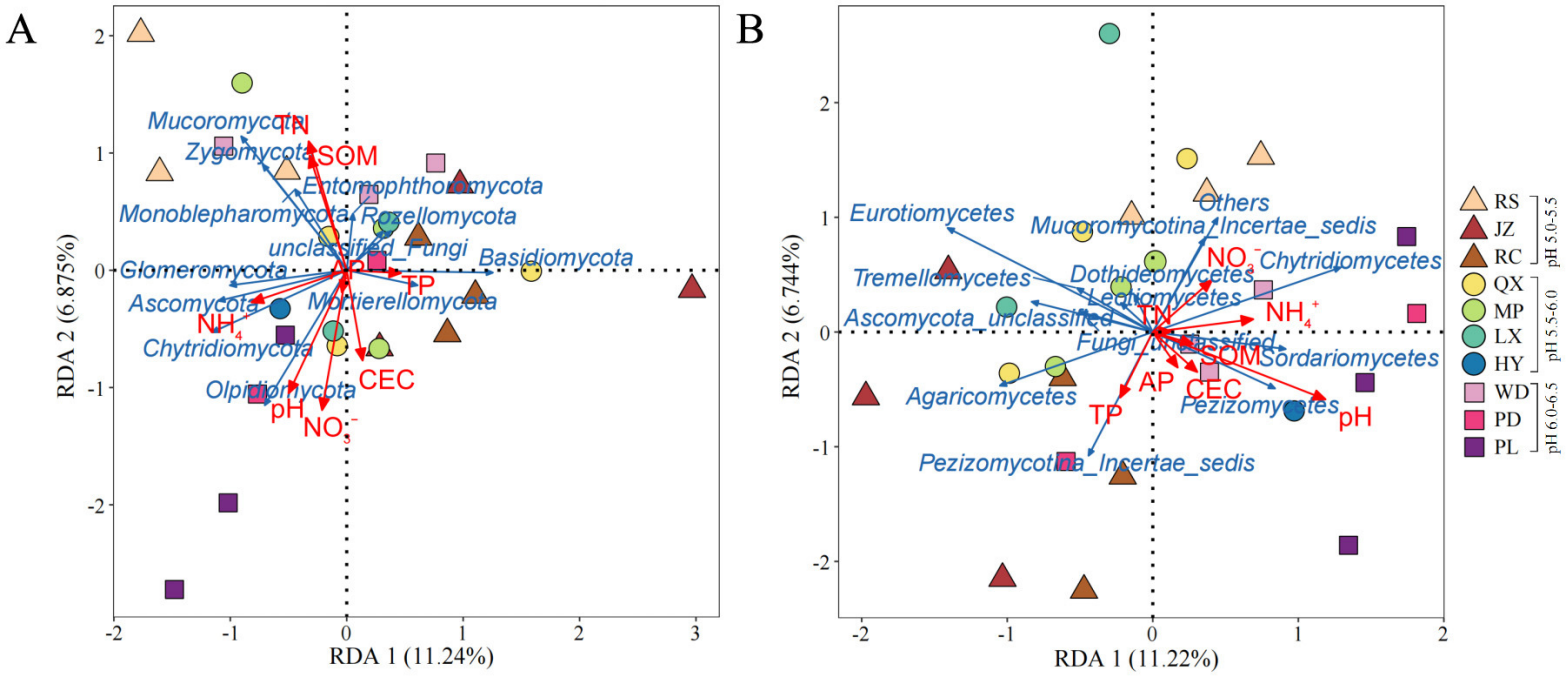
Redundancy analysis (RDA) of the relationships between fungal community structure at the **(A)** phylum level and **(B)** class level with soil environmental factors. The arrow length represents the strength of the correlation between the environmental factors and the fungi. And the acute angle (<90 degrees) represents a positive correlation, while the opposite indicates a negative correlation.

**Figure 7:**
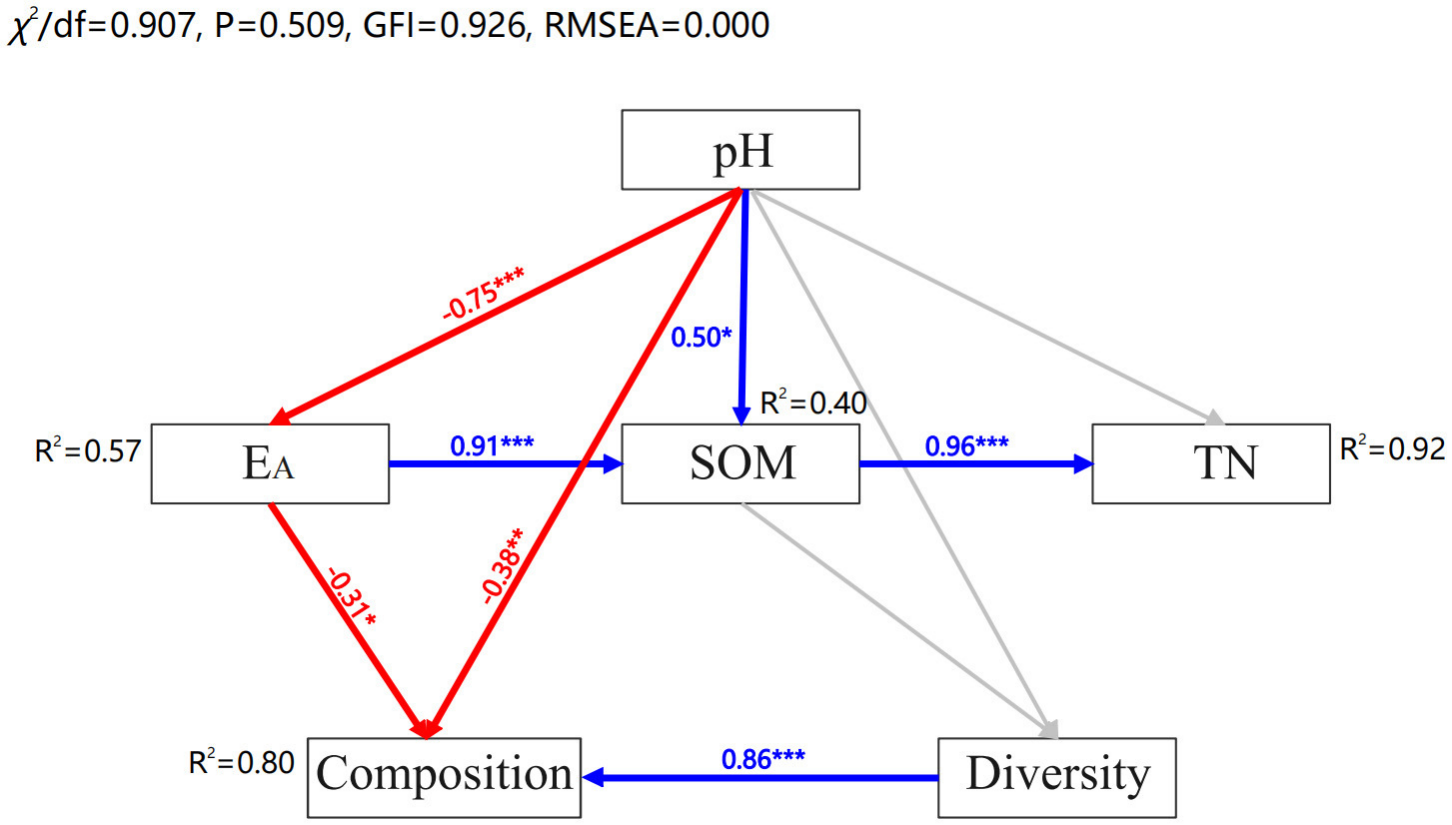
Structural equation modeling based on the effect of soil properties (pH, E_A_, SOM, and TN) on fungal diversity and composition. Blue and red arrows indicate significant positive and negative correlations, respectively. Gray arrows indicate non-significant relationships. Numbers at the arrows are standardized path coefficients. The width of the arrows indicates the strength of the relationship. *R*^2^ indicates the proportion of variance explained by the model. ***p < 0.001, **p < 0.01, *p < 0.05.

Redundancy analysis (RDA) was applied to identify the effect of environmental characteristics on soil fungal structure in the acidic soils (Figures 6A, B). The eight environmental factors (i.e., pH, SOM, TN, TP, ${\text{NO}}_{3}^{-}$-N, ${\text{NH}}_{4}^{+}$-N, AP, and CEC) explained 31.25 and 31.54% of the variation degree of fungal communities at phylum and class level in the acidic soil samples. The results showed that soil pH, ${\text{NO}}_{3}^{-}$-N and TN were significantly related to the changes in fungal community at phylum level (*p* < 0.05) (Figure 6A). And at the class level, soil pH had a significant impact on the variation of fungal community structure across all soils (*p* < 0.01) (Figure 6B). Additionally, structural equation modeling (SEM) was used to fit the causal relationships between soil chemical properties and fungal communities, which explained 80% of the community composition (Figure 7). It was indicated that the soil acidification indicators, including soil pH and E_A_ contents, had a negative direct influence on fungal community composition (*p* < 0.05). Overall, soil pH was the most important factor driving the soil fungal community composition.

### 3.4 Fungal functional guilds in acidic soils

**Figure 8:**
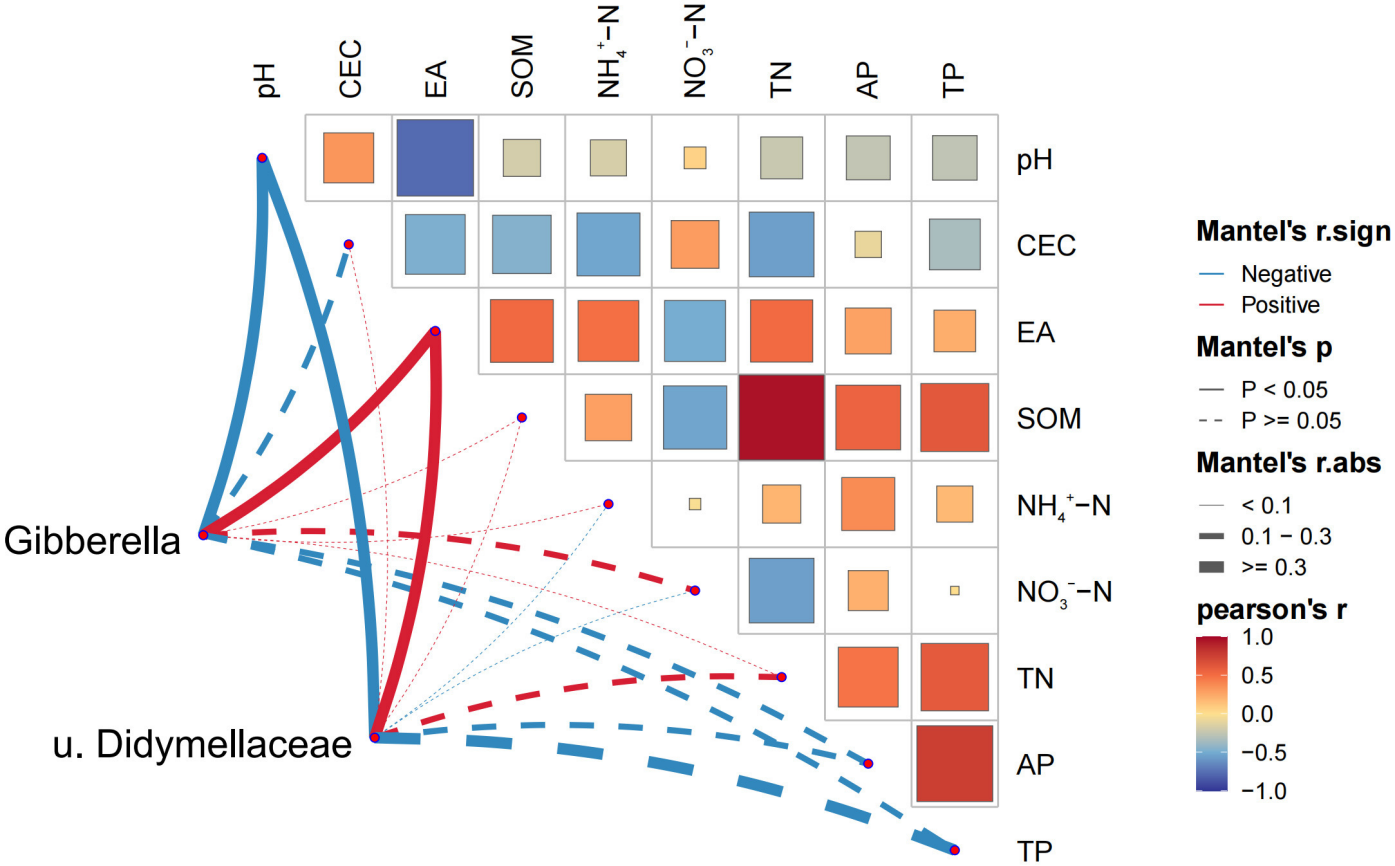
Correlation between *Gibberella* and unclassified *Didymellaceae* on soil environmental factors in Mantel test analysis.

According to FUNGuild database, 69% of fungi could be categorized into three trophic modes, including saprophytic, pathogenic, symbiotic, or a combination of these. Saprophytic fungi and pathogenic fungi were the most diverse vegetative flora, accounting for 66% of the total fungal communities. There were no significant differences in the spatial distribution of ASVs belonging to saprotrophs and symbiotrophs with soil acidic degree increasing (Supplementary Figure S5). However, compared to the soils with 6 < pH < 6.5, more ASVs were attributed to pathotrophs in soils with a pH ranging from 5 to 6. This result was consistent with the composition of fungal communities at the genus level. Further analysis of the relationship between enriched ASVs and environmental factors at the genus level, we selected *Gibberella* and unclassified *Didymellaceae* for correlation analysis with environmental factors. The ASVs assigned to *Gibberella* were identified as pathotroph, and which allocated to unclassified *Didymellaceae* were classified as pathotroph-saprotroph. The abundance of *Gibberella* and unclassified *Didymellaceae* significantly correlated with the values of pH and contents of E_A_ (*p* < 0.05) (Figure 8).

## 4 Discussion

### 4.1 Distribution and influencing factors of fungi in acidic soils of Jiaodong Peninsula

Fungal species are better adapted and tolerant to acidic environments than bacteria, and can live normally in soils with a pH range from 5 to 9 pH (Nevarez et al., 2009; Liu et al., 2015; Ye et al., 2020). Whilst, it was also reported that soil fungal diversity significantly declined with the decrease of soil pH primarily caused by the long-term N and P fertilizer application (Zhou et al., 2016). However, in our study, little difference in fungal diversity was observed despite of significant variations across soil pH gradients (Figures 2A, 3; Table 1). NMDS and ANOSIM results indicated little difference in soil fungal communities' composition existed across all the soil samples (Figure 4). The opposite results to the prior researches should be related to the wide acclimation of fungi to acidic soils (Xiao et al., 2018; Turley et al., 2020). In addition, it may also be ascribed to their similar soil management practices and cropping systems (Schmidt et al., 2019). In further, the above results might indicate a relatively stable agroecosystem in the Jiaodong Peninsula. However, although the results did not show significant differences in fungal diversity and composition along soil acidic degree, there were still unidentified fungi that have not been specifically analyzed. Furthermore, fungi are only a part of the agroecosystem which cannot represent the whole microbial system. To extend the validity of the findings of this study, further investigation including soil bacteria is required in a larger scale of soil samples.

In contrast to the similar taxa composition, a significant variation of the relative abundance for each fungal taxon, whatever at phylum, class or genera level, were found across various soil samples (Figure 5; Supplementary Figure S3), indicating that the composition pattern of soil fungal communities varied spatially in Jiaodong Peninsula. Similar to previous studies (Ye et al., 2020; Wang et al., 2022), *Ascomycota* and *Basidiomycota* were also identified as the dominant fungal phyla in acidic soils of Jiaodong Peninsula (Figure 5A). Variations in the relative abundance of *Basidiomycota* across sampling sites were consistent with soils acidification degree, whereas *Ascomycota* being more prevalent in soils with slightly acid (6 < pH < 6.5). *Ascomycota*, which accounted over 65% of total fungal communities, had significant saprotrophic capacity (Xiong et al., 2014; Maharachchikumbura et al., 2015). An in-depth analysis showed that species of *Pseudaleuria* (*Ascomycota, Pezizomycetes*), which were identified as ectomycorrhizal-undefined saprotrophs, were abundant in slightly acidic soils (Supplementary Figure S3) and facilitated plant uptake of nutrients (Hansen et al., 2013; Ma et al., 2023). This indicates that slightly acidic degrees are beneficial for *Pseudaleuria* growth in Jiaodong Peninsula, which in turn enhances soil productivity (Xiang et al., 2020). Furthermore, the *Podospora* (*Ascomycota, Sordariomycetes*) genus are considered to be antifungal agents (Che et al., 2002; Ding et al., 2017), which can suppress the number of pathogenic fungi (e.g., *Verticillium*) on plant disease (Xu et al., 2012). The relative abundance of *Podospora* in slightly and moderately acidic soils sampled from LX, HY, PD and PL was clearly higher than that in strongly acidic soils (Supplementary Figure S3). The LEfSe analysis showed that the enrichment of *Nectriaceae* existed in the pH 6.0–6.5 soils, while the pH 5.0–5.5 soils were enriched with *Didymellaceae*, suggesting that these fungal taxa could be potential biomarkers for these two acidic soil ecosystems, respectively (Supplementary Figure S4). Most members of *Didymellaceae* were plant pathogens with a broad host range, primarily causing lesions of the leaves and stems (Chen et al., 2017). However, the typical genera biomarker of *Nectriaceae* were belonged to saprotroph, which commonly occurred on the soil-litter interface and promoted soil organic C and N turnover and accumulation (Crowther et al., 2012; Yang W. et al., 2017; Zeng and Zhuang, 2022). Therefore, with soil acidic stress increasing, the changes of soil fungal taxa would cause a potential risk on soil nutrient cycling and crop health.

More and more studies suggested that soil microbial communities were sensitive to changes in soil physicochemical properties, including soil pH, SOM and N and P nutrients (Liu et al., 2020; Li B. B. et al., 2021; Liu et al., 2021). In this study, we also found that the contents of ${\text{NH}}_{4}^{+}$-N and ${\text{NO}}_{3}^{-}$-N were quite low in the strongly and moderately acidic soils sampled from RS, JZ, RC, QX, and MP (Figures 2C, D). That is, limited available N in acidic soils, fungi might need to allocate more energy for N uptake and assimilation, resulting in reduced biomass and metabolic rates (Li J. et al., 2021). The following RDA results found that, soil TN, ${\text{NO}}_{3}^{-}$-N as well as pH were significantly associated with fungal communities' abundance (Figure 6), indicating these factors played an important role on fungal community shifting across the investigated soils. This finding is similar to the findings of Dong et al. (2022) who found that mineral-N (${\text{NH}}_{4}^{+}$-N and ${\text{NO}}_{3}^{-}$-N) significantly affected the composition of the fungal communities. The further SEM analysis confirmed that soil pH played a crucial role in fungal community structure (Figure 7). This is consistent with the prior reports that soil pH was a master factor influencing the microbial community structure in acidic and neutral soils (Geisseler and Scow, 2014; Francioli et al., 2016; Ning et al., 2020). In general, the composition pattern and ecological function of soil fungal communities in agroecosystems of the Jiaodong Peninsula exhibited spatial variations, which mainly modulated by soil acidification.

### 4.2 Soil fungal functional groups in acidic soils and the potential ecological risk

The FUNGuild analysis revealed that the determined fungal communities in acidic soils of Jiaodong Peninsula mainly belonged to two functional groups of saprophytic and pathogenic fungi (Supplementary Figure S5). Among them, saprophytic fungi can convert complex SOM into available components (Francioli et al., 2021), while pathogenic fungi pose a serious threat to plant growth and health (Doehlemann et al., 2017), both of them are closely related to plant and soil productivity. Moreover, the distribution of saprophytic fungi showed no significant changes across three soil acidic degrees (strongly, moderately and slightly acid), indicating wide adaptations to the degraded environment (Boddy and Hiscox, 2016). Saprotrophic fungi preserved the capacity of soil nutrient supply under the stress of soil acidification (Pan et al., 2023). Whatever, the relative abundance of saprotrophs in slightly acidic soils were higher than that in strongly acidic soils, being consistent with the relative abundance of *Ascomycota*. It suggests that soil acidic stress might cause inhibition on the decomposition and transformation of organic material, yet affecting soil fertility in acidic soils (Ning et al., 2020).

The development and propagation of fungal plant pathogens in soil is a key factor stressing plant health and food security globally (Jones and Medina, 2020). The FUNGuild analysis indicated that plant pathogenic fungi constituted ~24% of the entire fungal community determined in soils of Jiaodong Peninsula, which might pose a potential threat to crop production as they include most of plant pathogens (Li Y. C. et al., 2020; Zhang et al., 2022). Moreover, the number of pathogenic ASVs in slightly acidic soils were lower than that in strongly acidic soils (Supplementary Figure S5). For example, *Gibberella* and unclassified *Didymellaceae*, the representative plant pathogenic fungi, were observed in soil samples of Jiaodong Peninsula, which had significantly higher abundance in strongly and moderately acidic soils (Supplementary Figure S3). *Gibberella* can easily lead to diseases such as root rot, crown rot, and wilting in crops (Gange et al., 1999; Kazan et al., 2012), indicating that the risk of root rot infection by *Gibberella* is higher in QX, MP, LX and HY than in other regions of the Jiaodong Peninsula (Tian et al., 2021). Interestingly, we found the highest relative abundance of unclassified *Didymellaceae* in strongly acidic soils sampled from RS, which could be affect photosynthesis and water transport in plants, thereby increasing the risk of disease in plants in RS. Moreover, the further analysis indicated that the abundance of *Gibberella* and unclassified *Didymellaceae* were positively correlated with soil acidification characteristics (Figure 8). Li et al. (2017) also showed that strongly acidic conditions favored the growth of the bacterial wilt in South China. Soil acidification greatly suppressed the capacity of soil microbiome to fight soil-borne pathogens (Li et al., 2023), seriously threating crop health. However, since some other factors (e.g., cropping systems, and MAT) were reported, which also affected the development of plant pathogens in agroecosystems (Thompson et al., 2014; Lu et al., 2022; Song et al., 2022), hence, the risk of infection by plant pathogens should be further evaluated in Jiaodong Peninsula. Taken together, functional guilds in the soils of Jiaodong Peninsula varied with soil acidic degree increasing, and these changes exerted adverse effects both on nutrient cycling and crop productivity.

## 5 Conclusion

In conclusion, our study demonstrated that in the Jiaodong Peninsula, soil fungal diversity exhibited no significant differences, but there is spatial variation in fungal community composition along the soil acidification gradient, yet there were all dominantly enriched with *Ascomycota* and *Basidiomycota* in acidic soils. Soil pH was identified to be the critical factor driving fungal community composition in acidic soils. LEfSe analysis in further revealed that the functional fungi contributing to soil organic matter degradation had a relative higher enrichment in the slightly acidic soils (6.0–6.5), while the fungi interacting with plant pathogens were well-enriched in the acidic soils (5.0–5.5). Therefore, soil acidification could cause more seriously potential risk on agroecosystem in addition to acidic soil environment. So that, in the future, more researches about acidic soils should be concentrated on the impact of microbes on the fertility and crop growth.

## Data Availability

The data that support the findings of this study are available from the corresponding author upon reasonable request. All the DNA sequence data in this manuscript are deposited in the NCBI Sequence Read Archive, accession number PRJNA1045351.
