# Supplementary material for: Composition and the predicted functions of fungal communities and the key drivers in acidic soils of Jiaodong Peninsula, China
Source: Front Microbiol. 2025 Jan 6;15:1496268. doi: 10.3389/fmicb.2024.1496268 (PMC11743958; doi:10.3389/fmicb.2024.1496268)
Supplement: Supplementary file 1 [file Data_Sheet_1.pdf]

Supplementary Material

Composition and the predicted functions of fungal communities and the key drivers in acidic soils of Jiaodong Peninsula, China

Jing Liu[1], Zafran Gul Wazir[1], Guoqin Hou[1], Guizhen Wang[1], Fangxu Rong[1], Yuzhi Xu[2], Kai Liu[2], Mingyue Li[2], Aiju Liu[2*], Hongliang Liu[3*], Hongwen Sun[4]

[1] School of Agricultural Engineering and Food science, Shandong University of Technology, Zibo, China

[2] School of Resources and Environmental Engineering, Shandong University of Technology, Zibo, China

[3] School of Life Sciences and Medicine, Shandong University of Technology, Zibo, China

[4] Ministry of Education Key Laboratory of Pollution Processes and Environmental Criteria, College of Environmental Science and Engineering, Nankai University, Tianjin, China

A

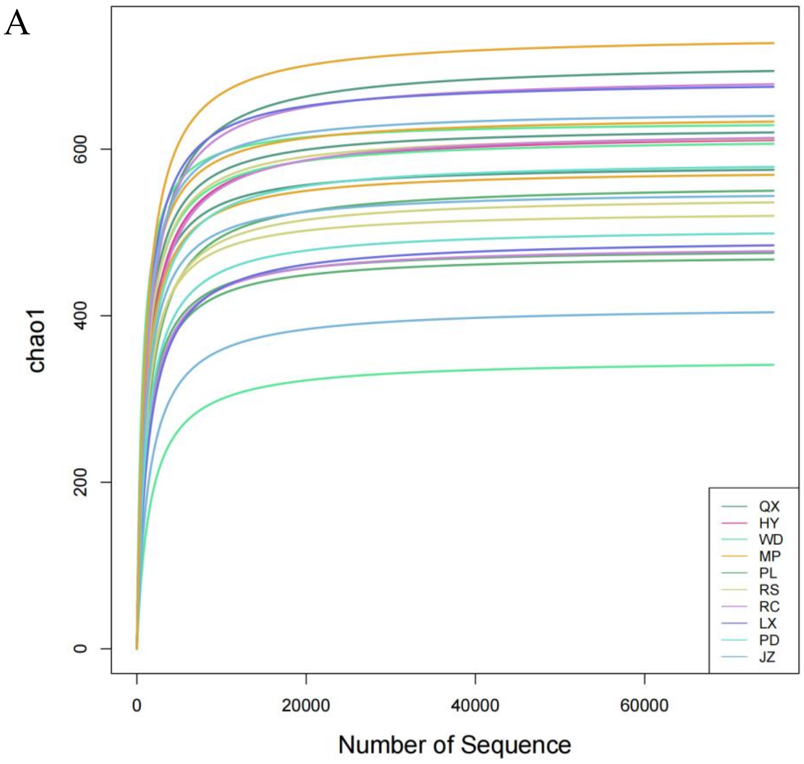

chao1
0
200
400
600
0
20000
40000
60000
Number of Sequence
QX
HY
WD
MP
PL
RS
RC
LX
PD
JZ

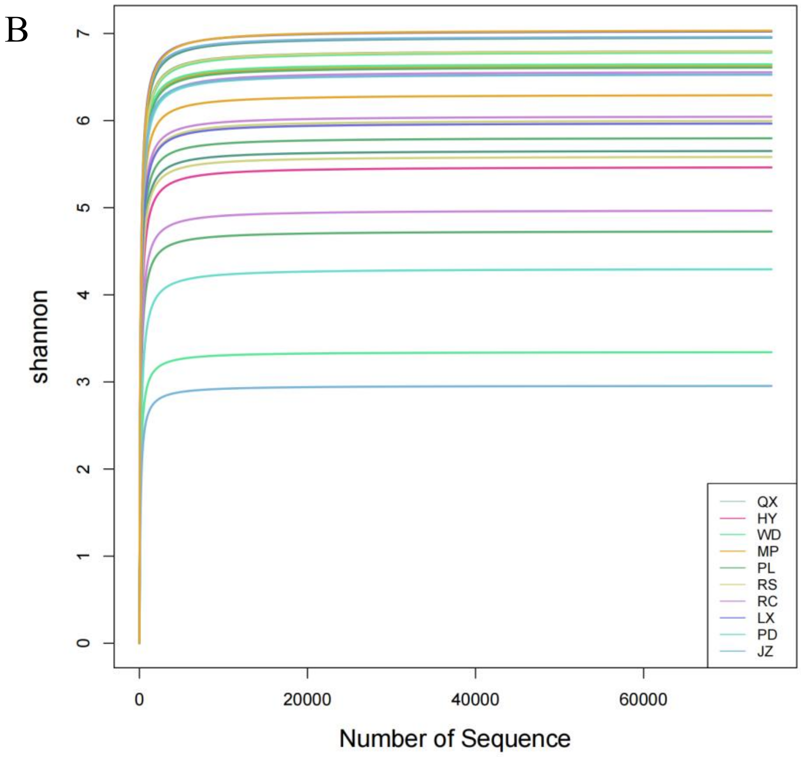

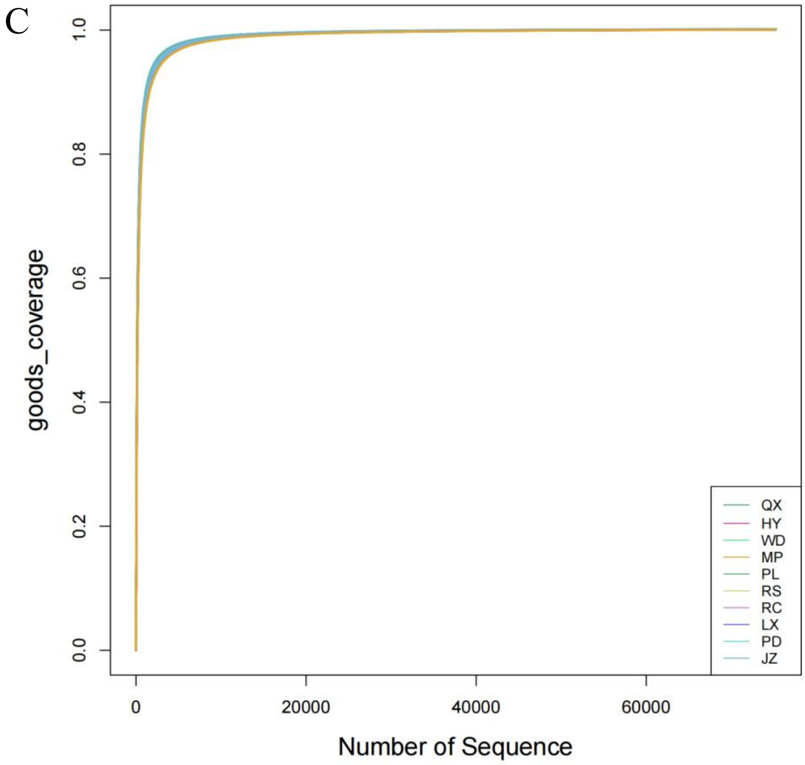

Supplementary Figure 1. The rarefaction curves of (a) Chao richness, (b) Shannon

index, and (c) Good's coverage at ASV level for the acidic soils.

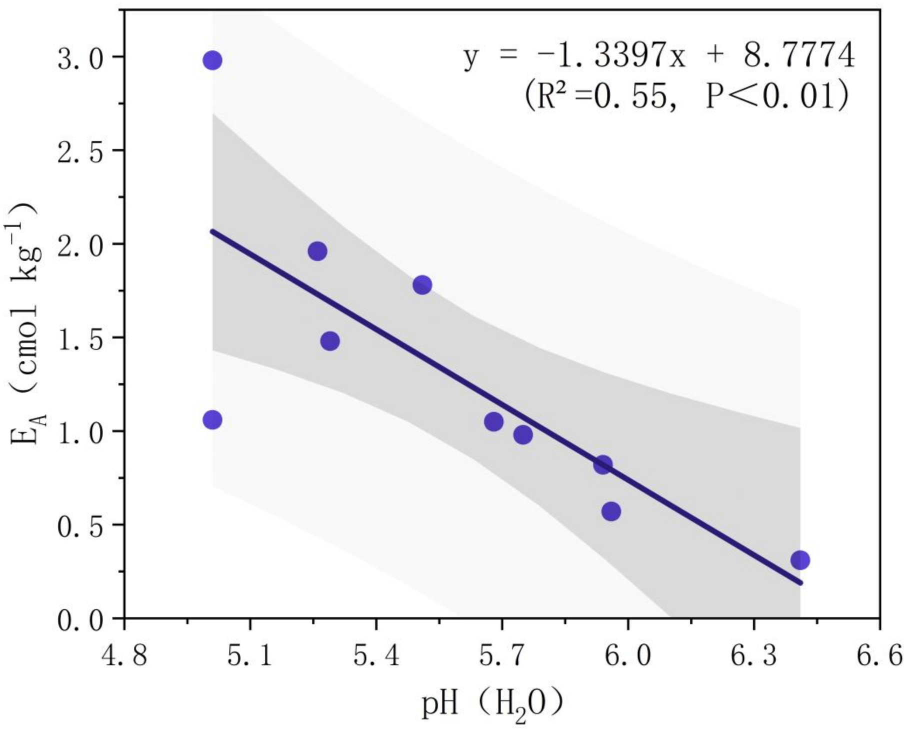

Supplementary Figure 2. Relationship between the contents of E_A relative to soil pH.

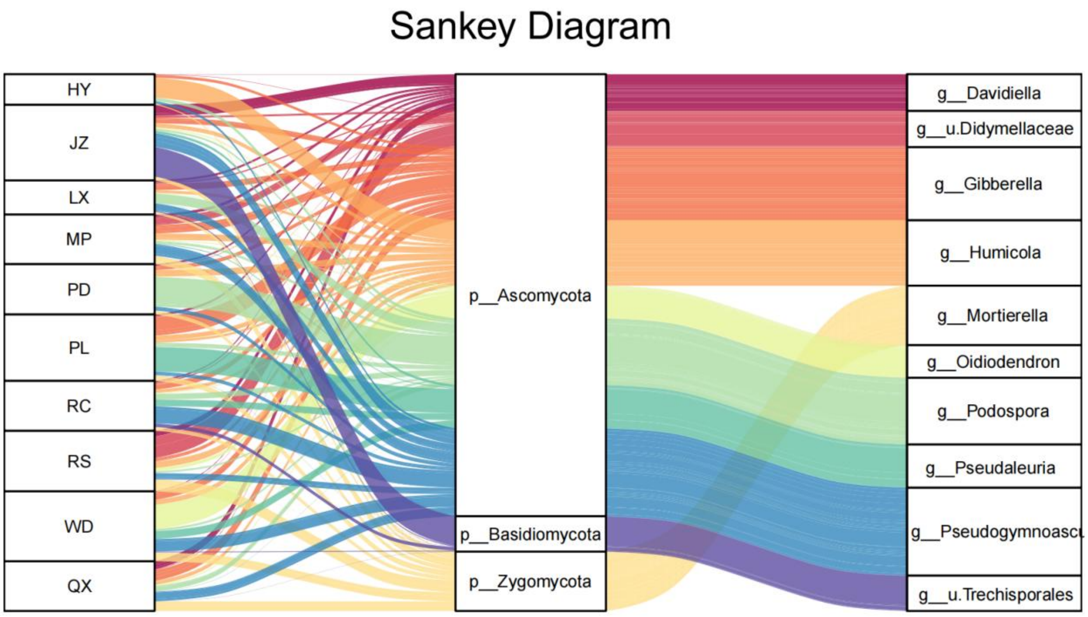

Supplementary Figure 3. Sankey diagram of soil fungal communities at ASV and phylum levels in Jiaodong Peninsula.

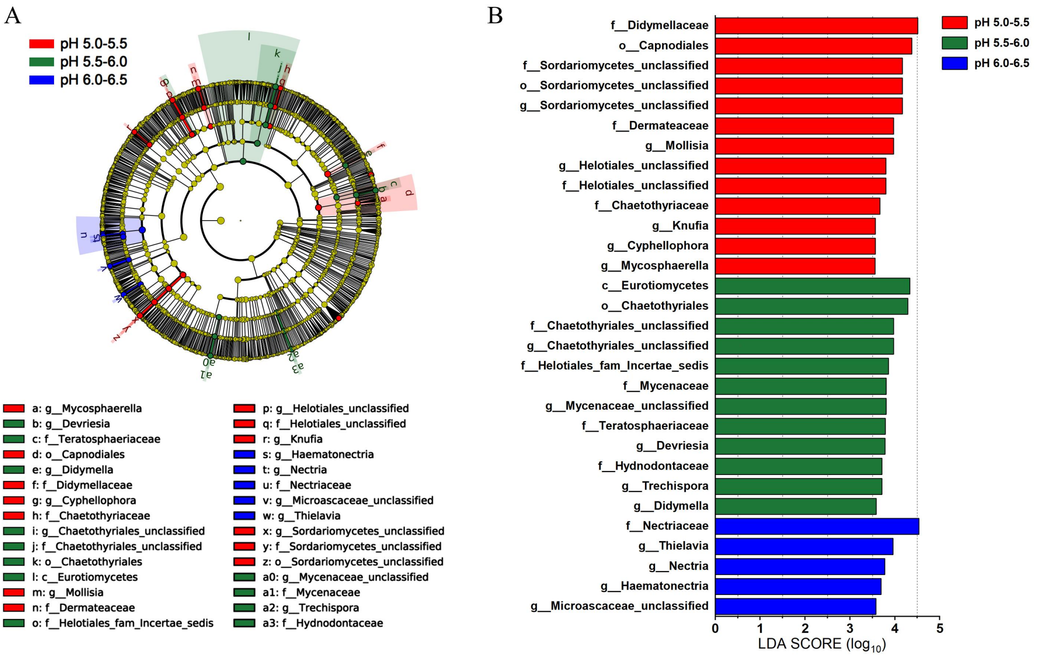

Supplementary Figure 4. LEfSe analysis of soil fungal abundance in croplands with different pH ranges: (A) LEfSe analysis results of soil fungi, and (B) Histogram of LDA scores calculated for the differentially abundant microbes with a threshold value of 3.5.

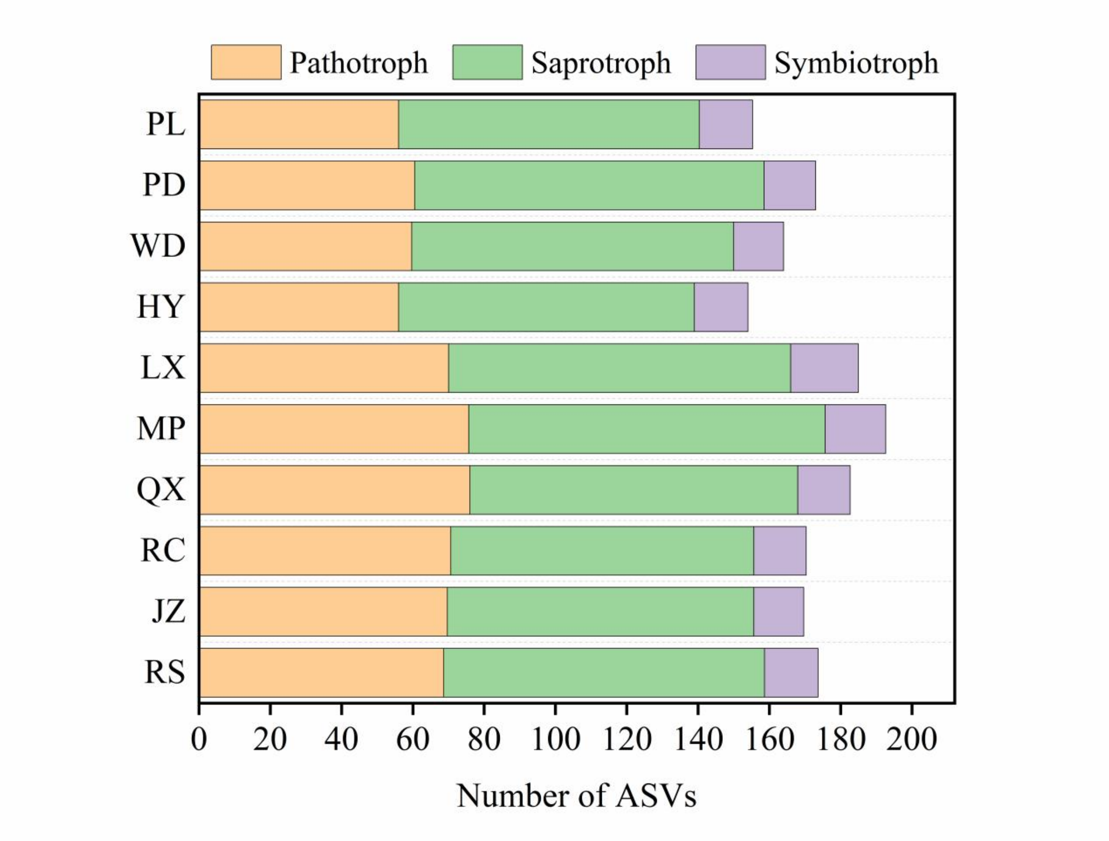

Supplementary Figure 5. Classification of fungal ASV functions in each soil sample.

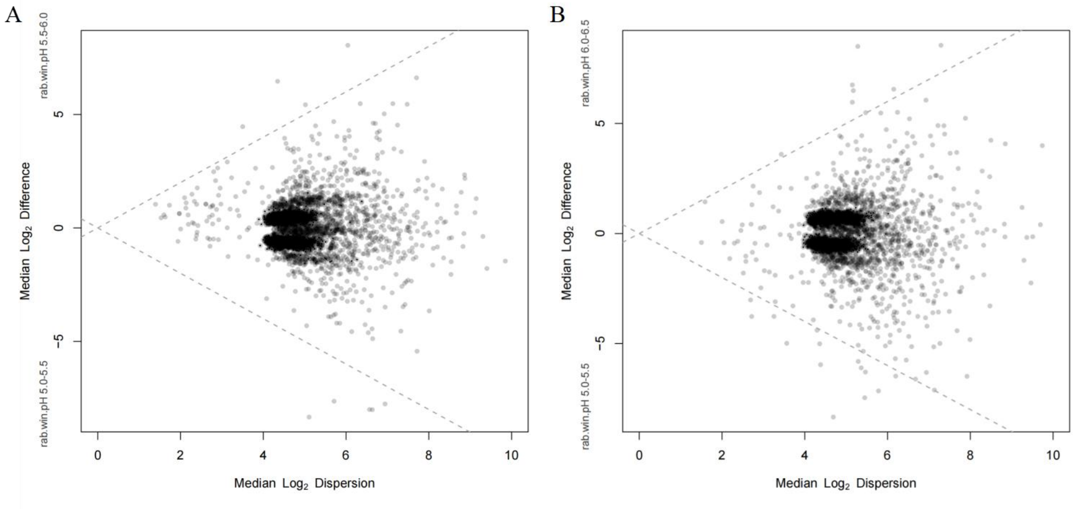

Supplementary Figure 6. ALDEx2 analysis of all soil fungi (A: pH5.0-5.5 vs pH5.5-6.0, B: pH5.0-5.5 vs pH6.0-6.5). Gray indicated taxa that were abundant but not significantly, while black indicated taxa with low relative abundance, also without significant difference (p > 0.05). The gray lines represented the equivalent lines of values within and between groups.
